# Sensitivity of Cancer Cells to Truncated Diphtheria Toxin

**DOI:** 10.1371/journal.pone.0010498

**Published:** 2010-05-05

**Authors:** Yi Zhang, Wendy Schulte, Desmond Pink, Kyle Phipps, Andries Zijlstra, John D. Lewis, David Morton Waisman

**Affiliations:** 1 Departments of Biochemistry and Molecular Biology and Pathology, Dalhousie University, Halifax, Nova Scotia, Canada; 2 Innovascreen Inc, Halifax, Nova Scotia, Canada; 3 Department of Pathology, Vanderbilt University, Nashville, Tennessee, Unites States of America; 4 Department of Oncology, University of Western Ontario, London, Ontario, Canada; Bauer Research Foundation, United States of America

## Abstract

**Background:**

Diphtheria toxin (DT) has been utilized as a prospective anti-cancer agent for the targeted delivery of cytotoxic therapy to otherwise untreatable neoplasia. DT is an extremely potent toxin for which the entry of a single molecule into a cell can be lethal. DT has been targeted to cancer cells by deleting the cell receptor-binding domain and combining the remaining catalytic portion with targeting proteins that selectively bind to the surface of cancer cells. It has been assumed that “receptorless” DT cannot bind to and kill cells. In the present study, we report that “receptorless” recombinant DT385 is in fact cytotoxic to a variety of cancer cell lines.

**Methods:**

*In vitro* cytotoxicity of DT385 was measured by cell proliferation, cell staining and apoptosis assays. For *in vivo* studies, the chick chorioallantoic membrane (CAM) system was used to evaluate the effect of DT385 on angiogenesis. The CAM and mouse model system was used to evaluate the effect of DT385 on HEp3 and Lewis lung carcinoma (LLC) tumor growth, respectively.

**Results:**

Of 18 human cancer cell lines tested, 15 were affected by DT385 with IC_50_ ranging from 0.12–2.8 µM. Furthermore, high concentrations of DT385 failed to affect growth arrested cells. The cellular toxicity of DT385 was due to the inhibition of protein synthesis and induction of apoptosis. *In vivo*, DT385 diminished angiogenesis and decreased tumor growth in the CAM system, and inhibited the subcutaneous growth of LLC tumors in mice.

**Conclusion:**

DT385 possesses anti-angiogenic and anti-tumor activity and may have potential as a therapeutic agent.

## Introduction

Diphtheria toxin (DT) is synthesized in *Corynebacterium diphtheriae* as a single-chain enzyme of 535 amino acids with a molecular weight of 63,000 [1], [2]. DT consists of three key domains: the amino-terminal C, or catalytic, domain (residues 1–186); the intermediate T, or transmembrane, domain (residues 202–381); and the carboxyl-terminal R, or receptor-binding, domain (residues 391–535). The catalytic domain is connected to the T domain by an arginine-rich loop and a readily reducible disulfide bridge (linking C186 to C201). DT has been shown to enter toxin-sensitive mammalian cells by receptor-mediated endocytosis which involves the interaction of the receptor-binding domain of the protein with a transmembrane cell surface precursor of the heparin-binding epidermal growth factor-like growth factor [3], [4]. After binding to this cell-surface receptor, DT is endocytosed and trafficked to an acidic vesicular compartment, where it undergoes a pH-dependent conformational change, cleavage and release of the catalytic domain. The T domain inserts into the vesicular membrane and the resultant channel is utilized for the translocation of the catalytic domain to the cytosol. There, the catalytic subunit catalyzes the ADP-ribosylation of elongation factor 2, resulting in the inhibition of protein synthesis and cell death (reviewed in [5]).

A number of truncated, recombinant DT proteins have been produced in which the receptor-binding domain has been genetically replaced by ligands that can selectively target malignant cells. These fusion proteins represent a novel class of cytotoxic agents which, unlike chemotherapeuticDT has been shown to enter toxin-sensitive mammalian cells by receptor-mediated endocytosis which involves the interaction of the receptor-binding domain of the protein with drugs, kill targeted cells by inhibiting protein synthesis and thereby inducing apoptosis[6]. These fusion proteins include DT508-MSF [7], DT486-IL-2 [8], DT486-GM-CSF [9], DT390-IL3 [10], DT388-GM-CSF [11]–[13], DT388-IL-3 [14], [15], DT385-VEGF [16], [17] and DT388 combined with the ATF domain of uPA [18]. Among the resulting drugs, DT388IL-3 has shown some promise in clinical trials [19], [20], whereas the DT389-IL-2 recombinant toxin (DAB389-IL-2, denileukin diftitox-Ontak) has been approved by the FDA for clinical use in advanced stage cutaneous T-cell lymphoma (reviewed in [21]–[23].

It is widely accepted that the efficacy of the DT fusion proteins lies in the ability of the targeting ligand component to direct the DT to cancer cells resulting in targeted cellular toxicity. Furthermore, the removal of the DT receptor-binding domain is expected to result in a truncated DT that is unable to interact with its receptor on the surface of eukaryotic cells and therefore unable to bind to and kill cells. This concept has been reinforced by the report that the truncated DT (DT385) is not cytotoxic [16].

In the current study, we show that contrary to previous reports, the recombinant truncated DT, DT385 is cytotoxic to many cancer cells. We also observed that DT385 inhibits the growth of human and mouse tumors. Our findings establish the efficacy of DT385 as a potential antitumor agent.

## Materials and Methods

### Cell Lines

Human Umbilical Vein Endothelial Cells (HUVEC) were obtained from Cell Applications, Inc. and grown in an endothelial cell growth medium with full growth supplements (Cell Applications, Inc.). Bovine pulmonary artery endothelial cells (BPAEC) and human dermal microvascular endothelial cells (HDMEC) were obtained from Lonza and were grown in an EBM medium plus EGM SingleQuots of growth supplements and EBM-2 medium plus EGM-2 SingleQuots of growth supplements (Lonza), respectively. Glioma cell lines U-87 MG and U251 were kindly provide by Dr. V. Wee Yong (University of Calgary, Calgary, Alberta, Canada). The human epidermoid carcinoma cell line HEp3 was a generous gift from Dr. Andries Zijlstra (Vanderbilt University, USA). Mouse embryonic fibroblast (MEF) cells were isolated from mouse embryos and were used at their early passages (less than passage 4). U-87 MG, U251, HEp3 and MEF cells were cultured in DMEM containing 10% (v/v) fetal bovine serum (FBS, Invitrogen) and 1% penicillin-streptomycin mixtures (Invitrogen). All other cell lines were obtained from American Type Culture Collection (ATCC, Rockville, MD) and were grown in DMEM, MEM or RPMI (Invitrogen) containing 10% (v/v) fetal bovine serum and 1% penicillin-streptomycin mixtures according to ATCC's instructions. All cells were grown in an incubator at 37°C containing 5% CO2. All endothelial cells and primary fibroblast cells were maintained and used before the ninth passage.

### Induction and purification of recombinant proteins

The plasmid pET17b-DT385 expressing “receptorless” DT385 was generously provided by Dr. Sundaram Ramakrishnan (Department of Pharmacology, University of Minnesota Medical School, Minneapolis, MN). The plasmid pET17b-p22 was constructed by cloning human plasminogen fragment p22 into pET17b (EMD Biosciences) at the NdeI and BamHI restriction sites. The plasmid pLIC-DT385-p22 was constructed by cloning human plasminogen fragment p22 into the plasmid pLIC-DT385 at the NcoI and HindIII restriction sites, while the plasmid pLIC-DT385 was constructed by inserting DNA encoding DT385 but lacking the stop codon into the pET-30 EK/LIC vector following the manufacturer's protocol (Novagen). The plasmid pSUMO encoding the cDNA for SUMO (small ubiquitin-related modifier, Saccharomyces cerevisiae, Smt3 gene) was kindly provided by Dr. Kaisong Zhou (Dalhousie University). All plasmid constructs were confirmed by DNA sequencing (DalGEN, the Dalhousie University DNA sequencing facility). Recombinant p22, SUMO, DT385, and DT-p22 were expressed in *E. coli* and purified by Ni-NTA affinity column (Qiagen), pooled and dialyzed overnight against phosphate buffered saline (PBS). All recombinant proteins were purified as a single band as revealed by SDS-PAGE analysis. LPS was removed from purified proteins using polymixin B beads (Detoxi-Gel Endotoxin Removing Gel, Pierce) according to the manufacturer's instructions. Multiple passes through the column were performed until LPS level was less than 30 EU/mg.

### Cell viability assay

Cell viability was assessed by the Cell Titer96 Aqueous One Solution Cell Proliferation Assay (MTS assay-Promega) using the manufacturer's protocol. The IC_50_ values were calculated by nonlinear least-squares fitting of dose-response curves using the open source computer program QTI PLOT. Data were analyzed with the four-parameter logistic equation f  = (a − d)/[1 + (x/c)b] + d, where *a* is the asymptotic maximum, *b* is a slope parameter, *c* is the value at the inflection point (IC_50_) and *d* is the asymptotic minimum. For a control, 0.4 µM or 1.2 µM DT385 was added to the tissue culture media in the absence of cells followed by incubation with the MTS/PMS reagent. Reduction of MTS was not observed under these conditions, confirming that DT385 did not interfere with this assay.

### Crystal violet stain

Cell proliferation was also evaluated with crystal violet staining. At the end of treatment with DT385, cells were fixed with 100% methanol, and stained with 0.5% crystal violet in 20% methanol for 15 minutes at room temperature. Stained cells were photographed at 25× magnification on a Zeiss Axiover 200 inverted microscope (Carl Zeiss, Germany).

### Apoptosis and Necrosis Assay

Apoptotic and necrotic cells were visualized with the apoptosis and necrosis assay kit (Biotium, Inc) following the manufacturer protocol. Stained cells were photographed on a Zeiss Axioplan II fluorescence microscope (Carl Zeiss, Germany), with appropriate filter settings for fluorescein isothiocyanate (FITC) and Texas Red fluorescence. Digital images were processed in Adobe Photoshop (Adobe Inc.).

### Protein synthesis assay

U-87 MG or Hela cells were seeded at a ratio of 1∶10 in 1 ml of DMEM plus 10% FBS per well in a 12-well plate overnight. Cells were treated with 1 µM DT385 for the indicated time, incubated for 15 minutes at 37°C with 0.5 ml labeling medium (methionine-free DMEM, 10% dialyzed FBS and 50–100 µCi Pro-Mix L-[^35^-S]-(Amersham)) and cell lysates analyzed by SDS-PAGE. Radioactive bands were visualized by radiography using a phosphorImager after overnight exposure on a phosphor screen. For quantitation, [^35^S] incorporation was measured by liquid scintillation counting.

### Chick chorioallantoic membrane (CAM) angiogenesis assay

The anti-angiogenic activity of proteins was tested on the CAM as described [24]. When analyzing tumor-induced vascularization, 50,000 HEp3 cells replaced bFGF/ VEGF as the angiogenic stimulus [25]. Four implants were placed on each CAM and ten embryos were used for each experimental compound.

### CAM tumor growth assay

HEp3-GFP tumour cells (100,000 cells/10 µl DMEM media) were applied directly to a filter-disc abraded area of the CAM of 9-day old chick embryos as detailed by [26]. Chicks were incubated under standard conditions until Day 15 when tumors were imaged and sorted for random distribution to control and treatment groups. Daily systemic intravenous injections of DT385 (2 µg in 50 µL PBS) or PBS (50 µL) were performed on day 15, 16 and 17. On day 18, tumors were excised, cleaned of CAM and then weighed. Alternatively, fluorescent (GFP) Hep3 tumors were imaged *in vivo* using a Zeiss Lumar fluorescence stereomicroscope. The outline of the tumor was determined using the fluorescence signal of the tumor cells (GFP channel (ex470/em525)). Specifically, Volocity software was calibrated to select areas with fluorescence intensity corresponding to 1 or more standard deviations above background. The area of the tumour was defined and calculated using Improvision Volocity software (Perkin Elmer, Inc.)(Version 5.3.0 Build 0). A unit of measure (1 mm) was calibrated using the grid of a hemocytometer. The determination of tumor volume assumes the shape to be hemiellipsoidal and the equation for the tumor volume is: V  =  π /6 • (length) • (width) • (height) where height  = 1.63√(Length •Width). Data were analyzed using Students t-test.

### Mouse tumor model

All animal work was carried out at the animal facility of Dalhousie University in accordance with the guidelines set forth in the Care and Use of Laboratory Animals by Dalhousie University. All investigations were approved by the Dalhousie Animal Research Ethics Board. Female 6-to 8-week old C57BL6/J mice (Jackson Laboratories) were used. Tumors were induced by subcutaneous injection of LLC cells (10^6^ cells) in 100 µl of sterile PBS. Palpable tumors were established three to four days after injection, at which point the mice were randomly assigned to two experimental groups, those that received recombinant SUMO (control) and those that received recombinant DT385 treatment. SUMO and DT385 were administered to mice, peritumorally at day 5 (25 µg in 100 µL PBS), and at days 9, 12 and 15 (10 µg in 100 µL PBS each injection).

### Protein Labeling with FITC

DT385 and BSA were FITC-labeled using the EZ-label FITC protein labeling Kit following the manufacturer's protocol (Pierce).

### Ammonium chloride washout studies

U87 cells were incubated in the absence or presence of DT385 (2 µM) alone or in combination with 10 mM ammonium chloride. The medium was removed at various time points and replaced with fresh media. Thirty six hours after the addition of test compounds, cell viability was measured by the MTS assay.

### Statistical Analysis

The significance of the data was determined using the Student's t-test (one-tailed). P values of <0.05 were regarded as significant.

## Results

### Characterization of DT385

**Figure 1:**
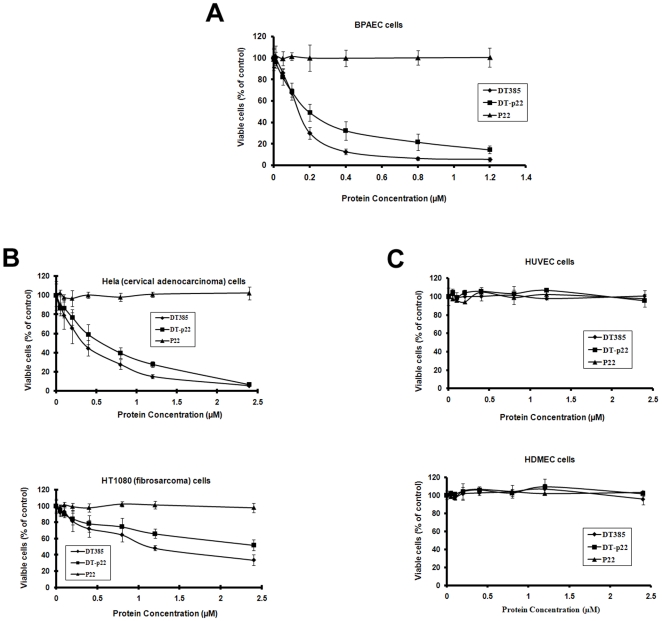
Effect of DT385 fusion protein and DT385 on cellular viability. Recombinant p22, (expressed from pET17b-p22), DT385 (expressed from pET17b-DT385), DT385-p22 (expressed from pLIC-DT385-p22), (see Materials and Methods) or an equivalent volume of PBS (control) were incubated with (A) BPAEC, (B) Hela and HT1080 cells, and (C) HUVEC and HDMEC cells in growth media for three days. After the three day incubation, viable cell numbers were quantified by the MTS assay. The PBS vehicle control was considered as 100% viable. Results are expressed as percentages of PBS-treated cells. Results are the mean ± S.D. of 3 independent experiments performed in triplicate (n = 9).

Recent studies from our laboratory identified and characterized a novel antiangiogenic fragment of plasminogen called p22 [27]. Since p22 was a potent and specific inhibitor of capillary endothelial cells, it was envisioned that this protein could be used to target DT to newly forming vasculature. We expressed a fusion protein in *E. coli* in which p22 was substituted for the receptor-binding domain of DT (DT385-p22). The activity of this fusion protein was compared with recombinant DT385 and recombinant p22. To determine the impact of these compounds on cell viability, MTS assays were performed on bovine pulmonary artery endothelial cells (BPAEC) after a three-day treatment. Interestingly, we observed that unlike the p22 prepared by proteolytic digestion of human plasminogen [27], the recombinant p22 failed to inhibit the viability of the BPAEC (Figure 1A). Unfortunately we have been unable to produce active recombinant p22 in *E. coli* or yeast (data not shown). Of particular interest was our observation that both the recombinant DT385-p22 construct and recombinant DT385 dramatically decreased the number of viable BPAEC. We determined an IC_50_ value of 0.21 µM and 0.14 µM for DT385-p22 and DT385 respectively (Table 1).

**Table 1:** Cytotoxicity of recombinant DT385.

Cell lines	IC50 (**µ**M)	Potency	Cell doubling time (h)
Human tumor or transformed cell lines			
U-87 MG	0.38±0.009	strong	**27 ^ref. 34^**
U251	0.46±0.0415	strong	40^**M**^
293T	0.12±0.027	strong	22 ^**ref. 33**^
HEK293	0.12±0.0085	strong	35^**M**^
Hela	0.33±0.013	strong	32^**M**^
Calu-3	0.13±0.022	strong	24^**M**^
Colo201	0.86±0.068	intermediate	
Colo205	0.87±0.059	intermediate	
LNCap	0.95±0.077	intermediate	
PC-3	0.98±0.11	intermediate	**24^M^/18 ^ref. 32^**
HT1080	1.21±0.09	intermediate	57^**M**^
MDA-MB-231 (1 treatment)	1.02±0.034	intermediate	43^**M**^
MDA-MB-231 (2 treatments)	0.66±0.024	intermediate	43^**M**^
MCF7	2.26±0.13	weak	16^**M**^
HCT116	2.82±0.28	weak	**17.7 ^ref. 31^**
NB4	ND	weak	
BT-20	ND	weak	
HL-60	ND	weak	
TIME	ND	weak	
HEp3 (1 treatment)	ND	weak	
HEp3 (2 treatments)	2.07±0.25	weak	
Human primary cell lines			
SC	ND	weak	
CCD-1064Sk	2.22±0.18	weak	
BJ	2.37±0.26	weak	
IMR-90	1.44±0.19	intermediate	
HUVEC	5.77±0.312	weak	**27 ^ref. 35^**
HDMEC	7.54±0.187	weak	
Bovine cell line			
BPAEC	0.135±0.0076	strong	
Mouse cell lines			
MEF	0.33±0.011	strong	
B16F10	0.463±0.011	strong	39^**M**^
LLC	5.42±0.29	weak	

Cells were treated with varying concentrations of DT385 for 3 days and cell viability was quantified by the CellTiter 96 Aqueous (Promega) assay as described in the legend to Figure 1. In order to determine the effect of double DT385 treatment, cells were treated with varying concentrations of DT385 for 2 days, washed with PBS and the procedure repeated for aCells were treated with varying concentrations of DT385 for 3 days and cell viability was quantified by the CellTiter 96 Aqueous (Promega) assay as described in the legend to Figure 1. In order to determine the effect of double DT385 treatment, cells were treated with varying concentrations of DT385 for 2 days, washed with PBS and the procedure repeated for another 2 days (2 treatments). To allow comparison of single and double treatments, one group of cells were incubated with varying concentrations of DT385 for 4 days (1 treatment).

IC_50_ is the concentration of DT385 reducing cell viability to 50% after 3 days. *Strong*: IC_50_ is less than 0.5 µM; *intermediate*: IC_50_ is between 0.5 µM–1.5 µM; *weak*: IC_50_ is greater than 1.5 µM.

*ND*: Not determinable, the inhibition of cell viability by compounds was less than 20% at the concentration of 2.4 µM.

*M*: measured in this study. Cells were grown in 96-well plates and measured daily by using the CellTiter 96 AQueous (Promega) assay. The doubling time of the cells was calculated from the exponential portion of the growth curve. nother 2 days (2 treatments). To allow comparison of single and double treatments, one group of cells were incubated with varying concentrations of DT385 for 4 days (1 treatment).

Since the recombinant p22 was inactive whereas DT385-p22 retained activity, it was unclear from these results if p22 might have retained activity when expressed as the fusion protein, DT385-p22. Considering the specificity of plasminogen-derived p22 for endothelial cells, we expected that if the p22 component of the DT385-p22 fusion protein, then DT385-p22 should only target endothelial cells and not cancer cells, as has been demonstrated for plasminogen-derived p22 [27]. We therefore tested the cytotoxic activity of DT385-p22 and DT385 with cancer cell lines. For these experiments we initially tested two commonly used human cancer cell lines, the HT1080 fibrosarcoma cell and the HeLa cervical carcinoma cell. Unexpectedly, we found that DT385 caused a dramatic loss in cell viability of the cancer cell lines in a dose-dependent manner (Figure 1B). At the highest dose tested (2.4 µM), 10% of viable Hela cells remained, while about 50% of viable HT1080 cells remained. DT385-p22 did also cause a loss in cell viability but was less potent than DT385. To determine whether the viability of human endothelial cells was affected by DT385-p22, we incubated HUVEC and HDMEC with these proteins (Figure 1C). Surprisingly, the viability of both cell lines was not affected by DT385, D385-p22 or p22 at the concentration up to 2.5 µM. We extended the incubation time to 7 days with 2.4 µM of these recombinant proteins, and no loss of cell viability was observed (Figure S1,A). However, with very high concentrations of DT385, loss of viability of the HUVEC and HDMEC was observed (Figure S1,B). We determined an IC_50_ of about 5.77 and 7.54 µM DT385 for the HUVEC and HDMEC, respectively. These results suggested that the cytotoxic activity of DT385-p22 was due to the activity of the DT385 domain of the fusion protein and not the p22 domain. We also concluded that DT385 can kill human cancer cells at a concentration not affecting primary endothelial cell lines. The observation that DT385 had cytotoxic activity was novel and unexpected. We therefore investigated the possibility that DT385 might kill other cancer cells.

### Cytotoxic effects of DT385 on cancer cell lines

We extended the cytotoxicity assay to 18 human cancer cell lines (Table 1). Fifteen cancer cell lines were affected by DT385 and efficacy of DT385 varied among the cancer cells. Cancer cell lines with highest sensitivity to DT385 (IC_50_ less than 0.5 µM DT385), included U-87 MG, U251, 293T, HEK293, Hela and Calu-3 cells. The group with intermediate sensitivity to DT385 (IC_50_ between 0.5-1.5 µM) included Colo201, Colo205, LNCap, PC-3, HT1080, and MDA-MB-231 cells. Weakly sensitive cancer cell lines with IC_50_ greater than 1.5 µM included MCF7, HCT116, BT-20, NB4, HL-60, and HEp3 cells. In contrast, recombinant p22 had no effect on cell viability at concentrations as high as 2.4 µM (data not shown). The three human cancer cell lines not affected by DT385 at the highest dose tested (2.4 µM) were the promyelocytic leukemia cell lines (HL-60 and NB4) and the epidemoid carcinoma cell line (HEp3).

**Figure 2:**
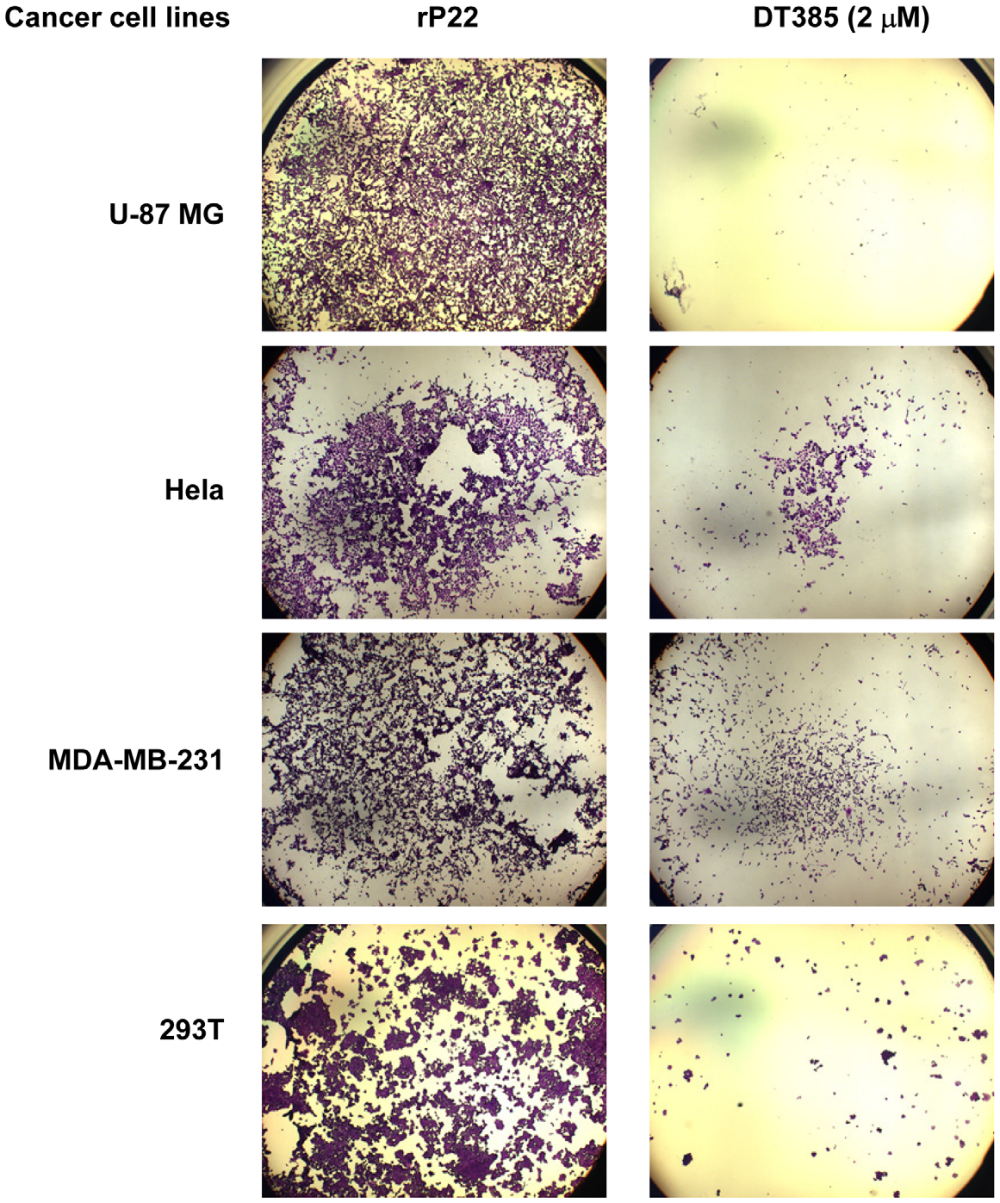
Cytotoxic effects of DT385 on cancer cell lines. Various cancer cell lines were cultured with 2 µM DT385 or recombinant p22 (rP22) for 3 days. Cells were then stained with crystal violet as described in Materials and Methods and images were obtained at 25× magnification with a Zeiss Axiover 200 inverted microscope (Carl Zeiss, Germany).

The DT385-mediated loss in cell viability was also confirmed using crystal violet staining (Figure 2). For example, less than 10% of glioma U-87 MG cells remained after DT385 treatment. To confirm cross-species sensitivity to DT385, we tested mouse skin melanoma (B16-F10), Lewis lung carcinoma (LLC) and mouse embryonic fibroblast (MEF) cells. While the B16-F10 and MEF were highly sensitive to DT385, the LLC were only weakly sensitive (Table 1). Together the data from MTS assays and Crystal Violet staining indicate that the “receptorless” DT, DT385, can indeed be cytotoxic to many tumor cell lines. The molecular mechanism that accounts for the broad range in sensitivity to DT385 is currently under investigation.

### Cytotoxic effects of DT385 on other primary cell lines

To evaluate the cytotoxicity of DT385 on other primary cell lines, primary cultures of three human fibroblast cell lines (CCD-1064Sk, BJ, and IMR-90) and one monocyte (SC) cell line were incubated with increasing concentrations of DT385 and the cell viability was determined by the MTS assay. CCD-1064Sk and BJ fibroblasts had an IC_50_ of 2.22 µM and 2.37 µM DT385, respectively. The lung fibroblasts (IMR-90) had an IC_50_ of 1.44 µM and the viability of the monocytes (SC) was unaffected by DT385 at concentrations up to 2.4 µM (Table 1). Again, recombinant p22 had no effect on cell viability at concentrations up to 2.4 µM (data not shown). In contrast, when confluent CCD-1064Sk, BJ and IMR-90 fibroblasts were treated for 3 days with 2 µM DT385, we observed no significant loss of cell viability (Figure S2). These data indicate that even when DT385 is effective in decreasing the viability of proliferating primary cells, it fails to kill these cells when they are quiescent/confluent. Taken together, these data support our previous conclusion that DT385 alone has the potential to kill cancer cells with minimum effects on primary cells.

To determine if the resistance to DT385 could be overcome by continuous incubation, cells with weak or no detectable sensitivity received a second dose, 48 hours after the first treatment and cell viability was measured 2 days later. The human epidermoid carcinoma cell line, HEp3 and breast cancer cell line MDA-MB-231 both exhibited enhanced sensitivity with continuous incubation with DT385. Compared to a single application of DT385, which did not affect HEp3 cells significantly, two applications of DT385 decreased the cell viability (IC_50_ of 2.07 µM (Figure S3). Similarly, the IC_50_ for the breast cancer cell line, MDA-MB-231 decreased from 1.02 µM to 0.66 µM upon a second incubation with DT385 (Table 1). In contrast, a second incubation with DT385 was without effect for the primary endothelial cells, HUVEC or HDMEC (Figure S3). Taken together, the results show that receptorless DT in the form of DT385 is cytotoxic to a large number of tumor cells. While the sensitivity to DT385 varies, continuous exposure diminishes the viability of even the most resistant cancer cell lines.

### Mechanism of action of DT385

**Figure 3:**
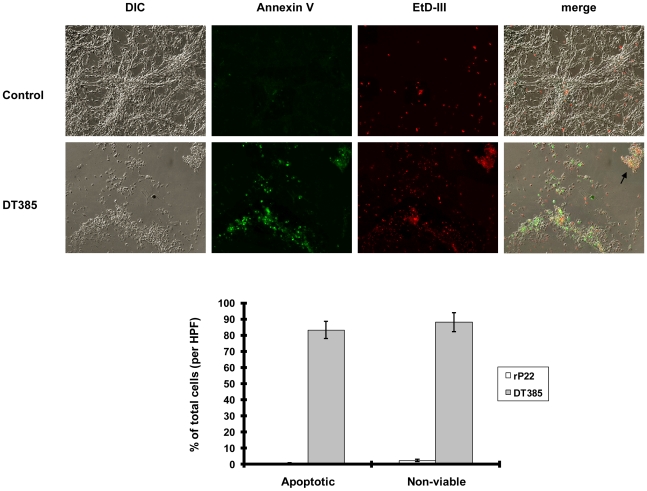
DT385 causes apoptosis and cell death in U-87 MG cells. (A), U-87 MG cells growing in an 8- well chamber slide were treated with 1.2 µM DT385, or control (PBS) respectively for 3 days. Following treatments, cells were stained for apoptosis with FITC-labeled annexin V. Cell membrane integrity was evaluated with ethidium homodimer III (EtD-III). Representative images (100× magnifications) are shown. The *arrow* indicates cells that were labeled with both fluorescent dyes. (B), Graphical representation of (A). The cell number per high-power field (HPF, ×400) was the mean of the cell number obtained from 5 random HPF. Detached cells, which were stained with both annexin V and EtD-III positive, were also included in the cell number determination. Results are the mean ± S.D. of 3 experiments.

DT kills cells by a mechanism involving cellular apoptosis. We also observed increased apoptosis in DT385 treated cells (Figure 3). The incubation of cultured U-87 with DT385 resulted in a dramatic increase in cellular apoptosis as measured by an increase in annexin V staining (85%) and the uptake of ethidium homodimer (87%).

**Figure 4:**
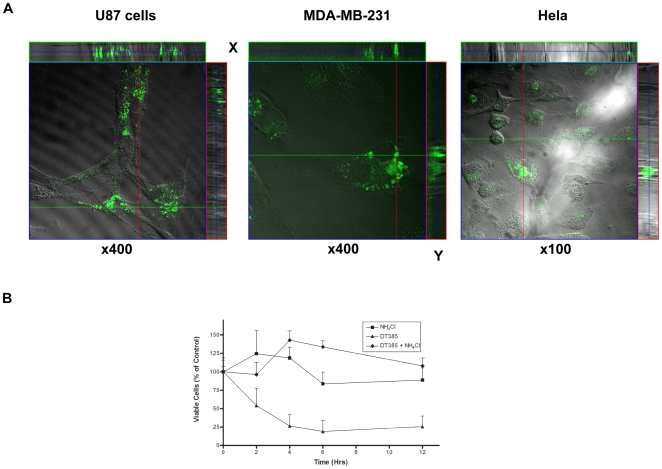
Internalization of DT385 by Cancer Cells. (A), Cancer cells were grown in an 8-well chamber slide. FITC-labeled DT385 (1 µM) was added to culture. The cells were observed and photographed under a Zeiss LSM 510 fluorescence confocal microscope (Carl Zeiss, Germany) after 36 h. Top and right sides of images demonstrates the orthogonal view of the confocal dataset shown in the x and y-axis of images. Magnifications are indicated below images. (B), U87 cells were incubated with 2 µM DT385 alone or in combination with 10 mM NH_4_Cl for the indicated times. The media was then replaced and cells were cultured for a total time of 36 hours after which cell viability was accessed with the MTS assay. The PBS vehicle control was considered as 100% viable. Results are expressed as percentages of PBS-treated cells. Results shown are representative of 2 independent experiments performed in triplicate.

DT has been shown to enter toxin-sensitive mammalian cells by receptor-mediated endocytosis which involves the interaction of the receptor-binding domain of the protein with its extracellular receptor. Since DT385 does not possess a receptor-binding domain, it should be incapable of binding to cells and becoming internalized. To investigate if DT385 was internalized, we tracked fluorescently labeled DT385 with confocal microscopy. Tumor cells sensitive to DT385 were incubated with FITC-DT385 and imaged with confocal microscopy. As shown in Figure 4A, incubation of cells with DT385 resulted in the internalization of the toxin which was detectable in perinuclear vesicles. In contrast, incubation of cells with similar concentrations of FITC-bovine serum albumin did not result in internalization of the protein (Figure S4). This experiment suggested that the uptake of DT385 by cells was not due to non specific cellular uptake of the protein.

Ammonium chloride is a weak base that diffuses into the endosome and serves as a proton reservoir, thus inhibiting the acidification of the endosome. We utilized ammonium chloride treatment of cells to investigate whether DT385 entered the cell by endocytosis. We observed that incubation of U87 cells with DT385 for as little as 2 hours resulted in significant cell death (Figure 4B). However, the simultaneous addition of ammonium chloride and DT385 abrogated the cytotoxic activity of DT385. The observation that ammonium chloride blocked the cytotoxic activity of DT385 suggests that DT-385 enters cells through an acidic endocytic pathway.

**Figure 5:**
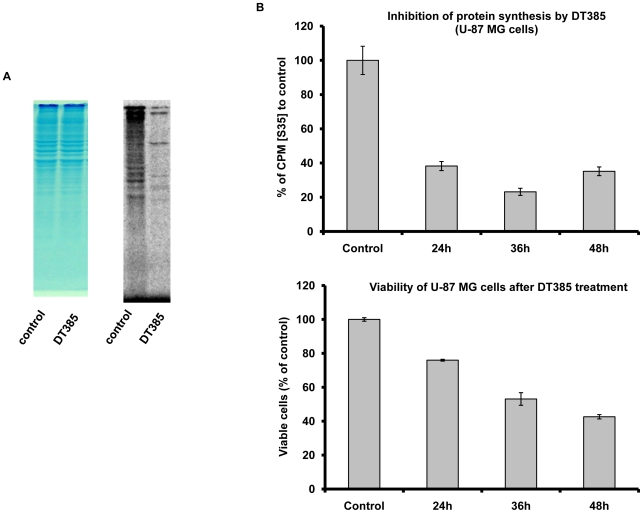
Inhibition of protein synthesis by DT385. (A), U-87 MG cells were treated with 1 µM DT385 or control (either rP22, SUMO or PBS) for 24, 36 h and 48 h, respectively, and then labeled with [^35^S-]methionine for 15 minutes. Cell lysates (10 µg) were separated by SDS-PAGE (12% gel) and gels were stained with Coomassie blue (left), dried on Whatman paper and visualized by radiography using a phosphorimager (right). Representative images for 36 h treatment are shown. (B), quantification of (A). Radioactivity of cell lysates was determined by liquid scintillation counting. Data are expressed as percent of control. The average of CPM from irrelevant protein, rP22 or recombinant SUMO or PBS treatment was considered as the control CPM value. Results are the mean ± S.D. (n = 6, 2 independent experiments). Cell viability was also measured as described in the legend to Figure 1. Results are the mean ± S.D. of three independent experiments performed in triplicate.

Diphtheria toxin kills cells by catalysing the ADP-ribosylation of EF-2, leading to inhibition of protein synthesis [5], [28]. To investigate if the DT385 decreased cell viability by inhibiting protein synthesis, glioma U-87 MG cells were treated with 1 µM DT385 for 36 hours followed by labeling with [^35^S-] methionine for 15 minutes. As shown in Figure 5A, SDS-PAGE analysis of cell lysate showed that protein synthesis was severely reduced in DT385 treated cells. This inhibition occurred within 24 hrs of treatment and persisted during the duration of the assay (48 hr) (Figure 5B). The 36-h treatment gave the greatest decrease in protein labeling. These data suggest that, mechanistically, DT385 decreases cell viability by blocking protein synthesis. Similar results were obtained for Hela cells (data not shown).

### Chick chorioallantoic membrane assay

**Figure 6:**
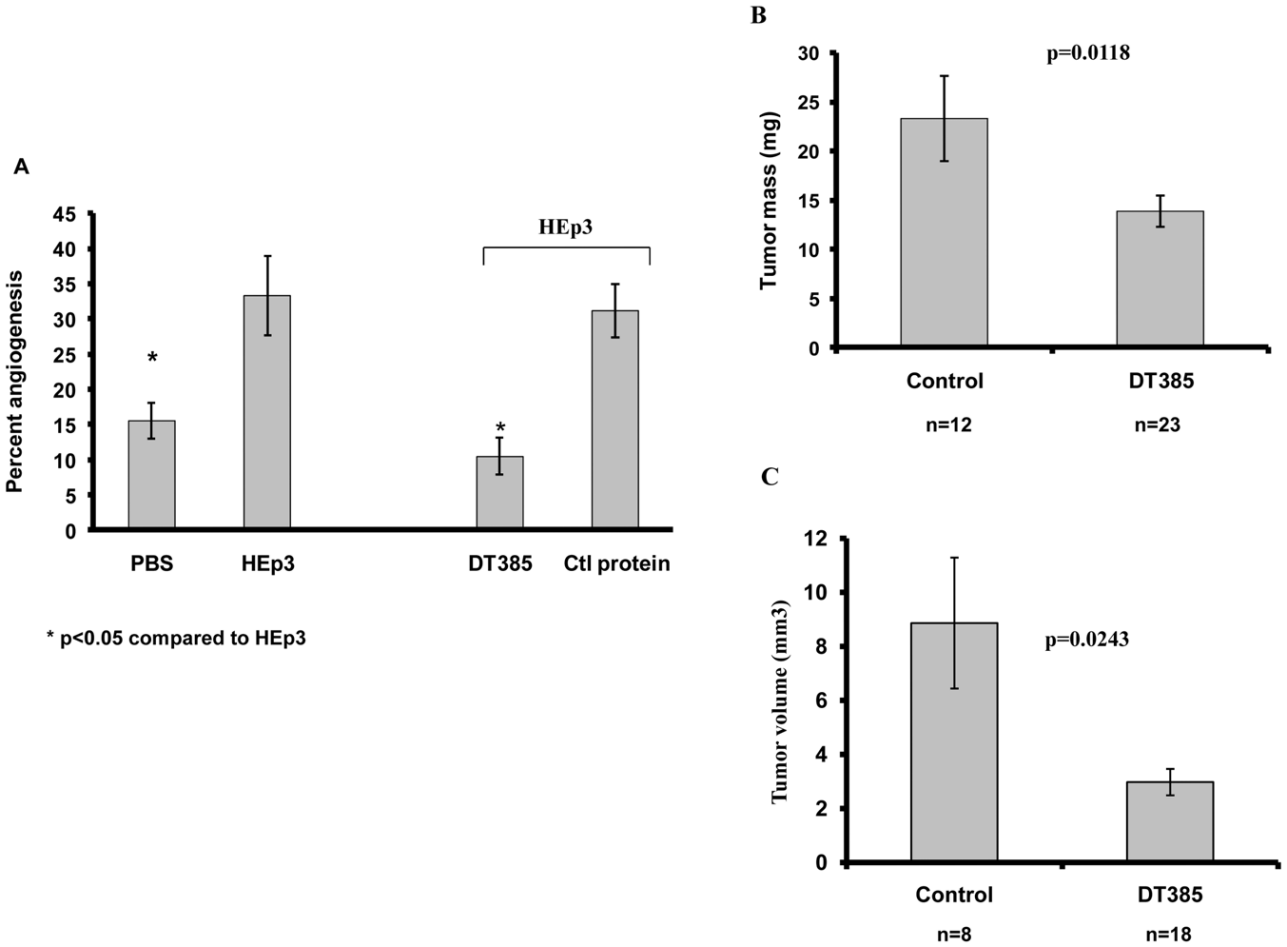
DT385 inhibited angiogenesis and reduce tumor growth in a chick chorioallantoic membrane model. (A), HEp3 cells were used to stimulate angiogenesis on the CAM. PBS control without HEp3 cells indicated the baseline level of angiogenesis. Angiogenesis, in the presence of HEp3 cells and in the absence or presence of recombinant DT385 or recombinant control protein (SUMO) was analysed. Results are the mean ± S.E. of 80 data points from two replicate assays *p<0.05 (Student t test). (B), DT385 significantly decreased tumor growth. Hep3 tumor growth in the CAM system was assessed as described in Materials and Methods. Results are the mean ± S.E. The mean values of tumors for DT385 or control treatments were 13.88±1.56 mg (mean ± S.E.; n = 23) and 23.33±4.3 mg, (mean ± S.E.; n = 12) respectively, p = 0.012. (C), DT385 significantly decreased tumor volume. Hep3 tumor volume in the CAM system was assessed as described in Materials and Methods. Results are the mean ± S.E. The mean values of tumor size for DT385 or control treatments were 8.86×10^9^±2.42 µm^3^ (mean ± S.E.; n = 18) and 2.97×10^9^±0.49 µm^3^ (mean ± S.E.; n = 8) respectively, p = 0.02.

The progression and metastasis of neoplastic disease requires extensive vascularization of the tumor to sustain the nutritional and oxygen demands of the proliferating cancer cells. This is generally achieved through a process called angiogenesis [29]. The chick chorioallantoic membrane assay (CAM) is a useful *in vivo* model system to investigate the effects of toxins on angiogenesis. As shown in Figure 6A, DT385 at a concentration of 40 nM (1.2 pmol in 30 µL) caused complete inhibition of HEp3-induced (angiogenic) vascular sprouting. This concentration is far below the IC_50_ of the tumor cells and therefore a direct effect of DT385 on HEp3 viability is unlikely.

To investigate whether DT385 could reduce tumor growth, HEp3 tumors were grown on the CAM and the 6-day old tumors were then treated with 2 µg of DT385 injected intravenously daily for three days. Assuming that the total blood volume of a 15–17 day chick embryo is approximately 2 mL [30], the initial systemic concentration of DT385 used in these experiments would be about 20 nM. Tumors were removed and weighed 24 hours after the last injection. We observed that DT385 decreased tumor mass by 41% (Figure 6B). We also observed a significant loss of 66% in tumor volume after treatment with DT385 (Figure 6C). These results indicate that DT385 can inhibit tumor angiogenesis and subsequently reduce tumor growth.

### Inhibition of tumor growth by DT385

**Figure 7:**
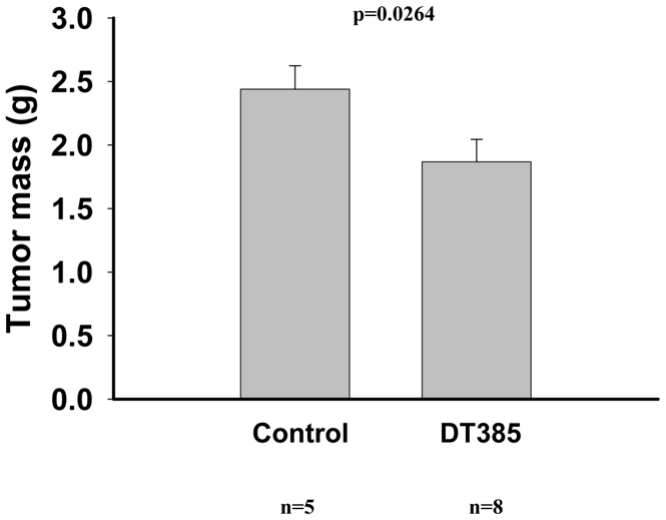
DT385 inhibited tumor growth in mice. Tumors were induced by subcutaneous injection of LLC cells (10^6^ cells). Recombinant SUMO (control) or recombinant DT385 were administered to mice, peritumorally at day 5 (25 µg), and at days 9, 12 and 15 (10 µg each injection). Tumor weights with the DT385 or control (SUMO) treatments were evaluated as described in the Materials and Methods. The mean values of tumors for DT385 or control treatments were 1.87±0.18 g (mean ± S.E.; n = 8) and 2.44±0.18 g (mean ± S.E.; n = 5), respectively, p = 0.0264).

To study the effect of DT385 in another *in vivo* model, LLC were implanted in mice and tumors were allowed to grow for a 19-day period. DT385 was administered subcutaneously on days 5, 9, 12 and 15 at a dosage of 25 µg for first injection and 10 µg for the remaining injections. We observed that tumor growth was significantly inhibited in mice receiving DT385 by day 19 (23%) when compared to control mice treated with an identical concentration of the recombinant control protein, SUMO (Figure 7). We did not observe any toxicity to the mice at these dosages. However, we observed an LD_50_ of 120 µg of DT385 (n = 10, data not shown) suggesting that higher doses of DT385 were lethal to mice.

## Discussion

The mechanism by which DT enters cells has been well established. The carboxyl-terminal receptor-binding domain (*R* domain-residues 391–535) interacts with the extracellular heparin-binding epidermal growth factor-like growth factor precursor protein and as a consequence the DT-receptor complex is internalized [3], [4]. Most cells possess this DT cell surface receptor and therefore DT has the capability of killing a wide range of cell types. However, it has been both assumed and also reported that the removal of the R domain renders the resultant truncated “receptorless” DT incapable of binding to and being internalized by cells. Previous studies had reported that the “receptorless” truncated DT was incapable of affecting cells [16]. Unexpectedly, we observed that the “receptorless” truncated DT, DT385, is capable of entering and killing a variety of cancer cells.

Interestingly, we observed that the efficacy of DT385 varied among the different cell types. While some cancer cells such as U-87 MG, U251, 293T, HEK293, Hela and Calu-3 cells had IC_50_ values less than 0.5 µM, other cancer cells Colo201, Colo205, LNCap, PC-3, HT1080, and MDA-MB-231 cells has an intermediate sensitivity to DT385 (IC_50_ between 0.5–1.5 µM). Some cancer cell lines including MCF7, HCT116, BT-20, NB4, HL-60, and HEp3 cells had IC_50_ values greater than 1.5 µM and others were unaffected by DT385 at the concentrations examined. Although speculative, it is possible that the diversity in efficacy to DT385 demonstrated by cultured cells is due to either differential rates of inactivation of DT385 by the cells or by differences in the uptake of the toxin. We have shown that DT385 is internalized by cells (Figure 4A), by an endocytotic mechanism (Figure 4B).

It was also interesting that primary cells and primary cell lines were generally much more resistant to DT385 than cancer cells. We initially considered the possibility that the efficacy of DT385 might be a function of the cell doubling time. However, this was not true. For example, 293T, PC-3 and HCT116 are highly, intermediately and weakly sensitive to DT385, respectively. However, the growth rate of these cells is similar. The doubling time is ∼17.7 h for the HCT116 cells [31] ∼18 for PC-3 cells [32], and 22 h for 293T cells [33]. The glioma cell line U-87 MG is very sensitive to DT385, whereas human endothelial cell line HUVEC is not affected by DT385 up to 2.5 µM. These two cell lines have the same doubling time of ∼27 h [34], [35]. We measured the growth rate of 9 cell lines, but found no correlation between the cell growth rate and the sensitivity to DT385 (Table 1). We also observed that confluent cells were resistant to DT385. Confluent cells are known to have greatly reduced rates of protein synthesis [36]. Therefore, it is likely that the reduced rates of protein synthesis observed with the confluent cells are responsible for the ineffectiveness of DT385.

It is interesting to note that human endothelial cells are resistant to DT-385 treatment *in vitro* but angiogenesis is inhibited in CAM assays *in vivo*. The endothelial cells are not the only cells that are involved in angiogenesis in the CAM system. The seemingly conflicting data is possibly the result of an effect of DT-385 on chicken inflammatory cells, as these are critical for a robust angiogenic response. Alternatively, our observation that bovine pulmonary artery endothelial cells are sensitive to DT385 suggests that the species of the endothelial cell may be a factor in its sensitivity to DT385.

It has been reported that one molecule of DT introduced into the cytosol of a cell is sufficient to cause cell death [37]. Since one DT molecule catalyses the ADP-ribosylation of 2000 EF-2 molecules/min *in vitro* [38], and since one cell contains about 10^6^ EF-2 molecules [39], it can be roughly estimated that once a single DT molecule has entered the cytoplasm it would require about 30 h to inactivate all the EF-2 molecules in a cell [40]. Consistent with this calculation, we observed that the inhibition of protein synthesis peaked 36 hours after exposure to DT385. We also used FITC-labeled annexin V to detect apoptosis and ethidium homodimer III (EtD-III) to examine the loss of cell membrane integrity. We observed that maximal apoptosis and loss of membrane integrity occurred after 72 h treatment (Figure S5).

The potential usefulness of DT385 as an antitumor agent was evaluated in both the chick CAM assay (Figure 6) and the Lewis lung carcinoma mouse model (Figure 7). Although, we observed a significant reduction in tumor mass in both *in vivo* systems, the reduction of the mouse tumors was not dramatic. However, the mouse LLC cells used in these studies are among the least sensitive cells to DT385. Extensive experimentation involving testing the modality and frequency of injection of DT385, as well as the concentration will be required before the potential efficacy of DT385 as an antitumor agent can be fully appreciated. It is also possible that combining DT385 with other chemotherapeutic drugs may increase the efficacy of the toxin. Considering the high sensitivity of glioma cell lines to DT385, we cannot rule out the possibility that certain forms of cancer may be very sensitive to DT385. For example, glioblastoma is form of astrocytic brain tumors that is refractory to chemotherapy in most cases. We have observed that U-87 MG cells, which are used as an *in vitro* model of human glioblastoma are among the most sensitive cells to DT385 (Table 1). It is therefore possible that DT385 alone or in combination with other anti-tumor drugs might prove effective against this form of cancer. Taken together, our data directly demonstrate that DT385 is a cytotoxin capable of killing tumor cells and arresting tumor growth. Although we observed that DT385 killed cultured cancer cells due to its ability to inhibit protein synthesis, we cannot rule out the possibility that the toxic effect of DT385 on tumor growth in our zenograft (Figure 6) and ectopic models (Figure 7) was not due to protein synthesis inhibition.

Our report has potentially important implications as to the specificity of the fusion proteins that are currently being tested as anticancer agents. It is currently believed that fusion proteins such as DT388IL-3 and DT389-IL-2 (denileukin diftitox-Ontak) recombinant toxin that are used in the treatment of lymphoma derive their specificity by virtue of targeting the toxin to IL receptors. However, our data suggests that at sufficiently high concentration of toxin, cells lacking IL receptors may be non-specifically targeted by the DT domain of the fusion protein. Although at the low concentration of these toxins that are currently deployed in treatment schemes, it is unlikely that non-specific cytotoxic effects will occur, we cannot rule out the possibility that at higher concentrations and with repeated doses of these toxins, non-specific cytotoxic effects involving other cells or tissues might occur.

Considering the prevalence of childhood vaccination for DT, the truncated native protein might be subject to that established immune response. While the native protein may not be the ideal mechanism for clinical implementation, truncated DT could be the basis for a functional anti-tumor strategy. Further modification of the protein will be evaluated with the objective in mind of minimizing its antigenicity and avoiding the established immune protection in immunized individuals. Furthermore, ongoing work in drug delivery systems such as liposomes may offer an effective mechanism to shield truncated DT from the immune system while on route to the tumor tissue.

The current strategy of intratumor injections provide an effective proof of principle. Currently, intratumoral injections have been shown to be useful for a limited number of tumors such as malignant glioma. Future clinical implementation will require systemic delivery. Fortunately, recent advances in the field of cancer therapeutics will likely make it possible to package and deliver anti-cancer therapeutics systemically.

## Supporting Information

Figure S1 Effect of DT385 on human endothelial cells. A), Time response curves. Cells were treated with 2.4 µM of either recombinant p22, DT385 or DT385-p22, for 7 days. Viable cells were determined at indicated time points as described in the legend to Figure 1. Media were replaced every 3 days. B), Dose response curves. The concentration of DT385 was increased to 10 µM, and cell viability was measured 72 h later as described in the legend to Figure 1. Data are expressed as percent control response versus incubation time. Results are the mean ± S.D. of 3 experiments performed in triplicate.
(0.17 MB TIF)

Figure S2 DT385 did not kill confluent cells. Cells were grew to confluent first, then incubated in fresh media and treated with 2 µM of DT385 for 3 days. Viable cell numbers after the three day incubation were quantified by the CellTiter 96 AQueous (Promega) assay. Cells without treatments were used as controls. Alternatively, PBS or control protein treatments were used as controls. Data are expressed as percent control response. Results are the mean ± S.D. of 3 experiments performed in duplicate (n = 6).
(0.44 MB TIF)

Figure S3 Two Applications of DT385 and DT-p22 Increased the Proliferation Inhibition Effect on Tumor Cells, but not on Human Endothelial Cell Lines. Cells were first treated with DT385 for 48 h, washed with PBS and then were further treated with a second application of DT385 for 48h (2 treatments) or simply incubated with DT385 for 4 days (1 treatment). Viable cells were then quantified by the CellTiter 96 AQueous (Promega) assay. A), Hep3, B), MDA-MB-231 cells were administrated DT385 at the indicated concentration. C), HUVEC or HdMEC cells were administrated 2 applications of DT385 at a concentration of 2.5 µM. PBS was used as a control treatment. Data are expressed as percent of control treatments. Results are the mean ± S.D of 2 experiments performed in triplicate (n = 6).
(0.20 MB TIF)

Figure S4 Internalization of DT385 by Cancer Cells. Cancer cells were grown in an 8-well chamber slide. FITC-labeled DT385 or BSA (DT385-FITC or BSA-FITC, 1 µM) was added to culture. The cells were observed and photographed with a Zeiss Axioplan II fluorescence microscope (Carl Zeiss, Germany) after 36 h. A), bright-field images. B), fluorescence images.
(3.81 MB TIF)

Figure S5 Time Course Assays of Apoptosis Caused by DT385. U-87 MG cells growing in an 8- well chamber slide were treated with 1.2 µM of DT385 (A) or control protein (B), respectively. Following treatments, cells were stained for apoptosis with FITC-labeled annexin V. Cell membrane integrity was evaluated with ethidium homodimer III (EtD-III) according to the manufacturer's instructions (Biotium, Inc). Staining images were photographed under a Zeiss Axioplan II fluorescence microscope (Carl Zeiss, Germany). Digital images were processed in Adobe Photoshop (Adobe Inc.). Representative images (100× magnifications) were shown.
(7.69 MB TIF)
