# Supplementary figures and images for: Sensitivity of Cancer Cells to Truncated Diphtheria Toxin
Source: PLoS One. 2010 May 5;5(5):e10498. doi: 10.1371/journal.pone.0010498 (PMC2864767; doi:10.1371/journal.pone.0010498)

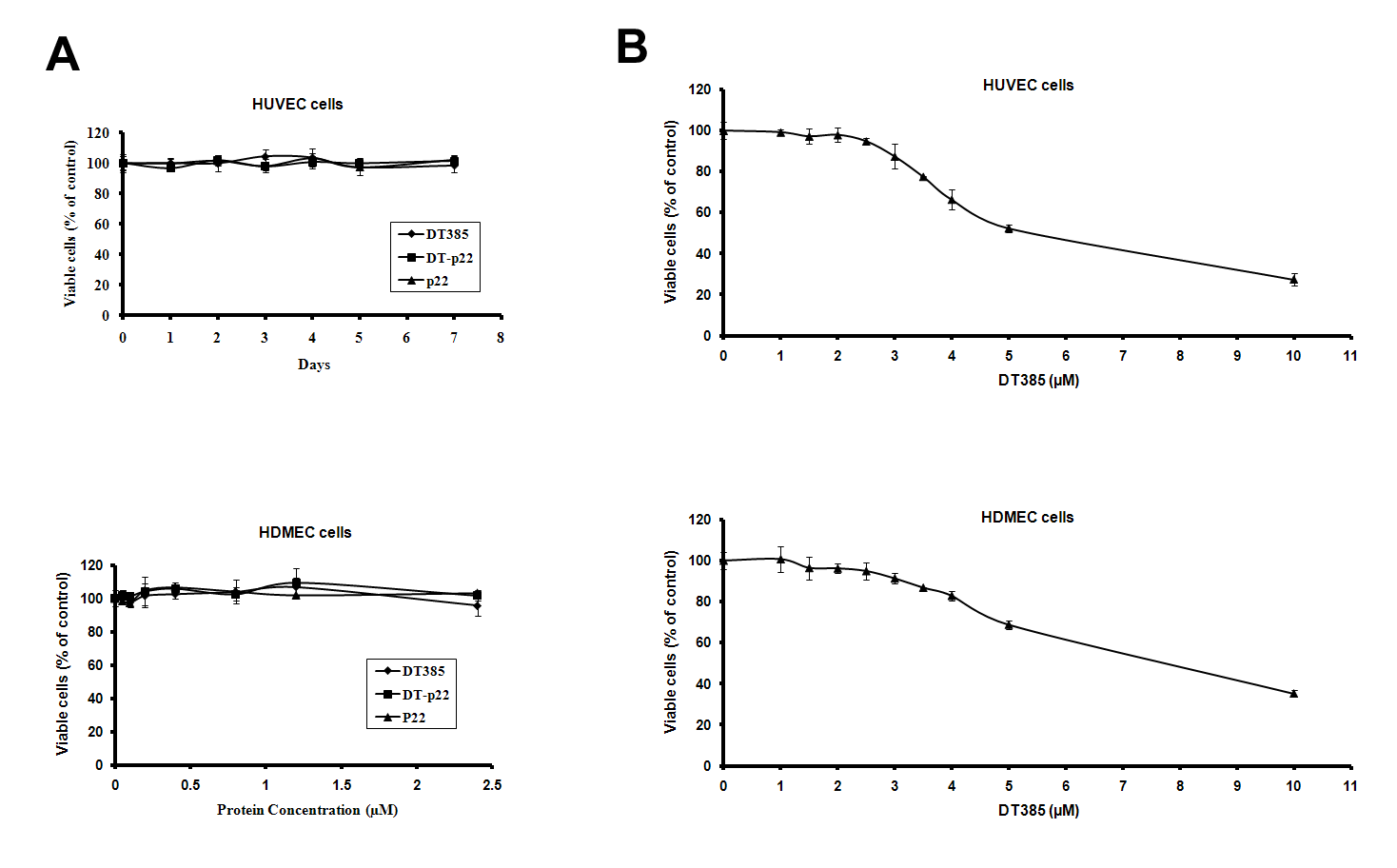

Supplement: Figure S1 — Effect of DT385 on human endothelial cells. A), Time response curves. Cells were treated with 2.4 µM of either recombinant p22, DT385 or DT385-p22, for 7 days. Viable cells were determined at indicated time points as described in the legend to Figure 1. Media were replaced every 3 days. B), Dose response curves. The concentration of DT385 was increased to 10 µM, and cell viability was measured 72 h later as described in the legend to Figure 1. Data are expressed as percent control response versus incubation time. Results are the mean ± S.D. of 3 experiments performed in triplicate. (0.17 MB TIF) [file pone.0010498.s001.tif]

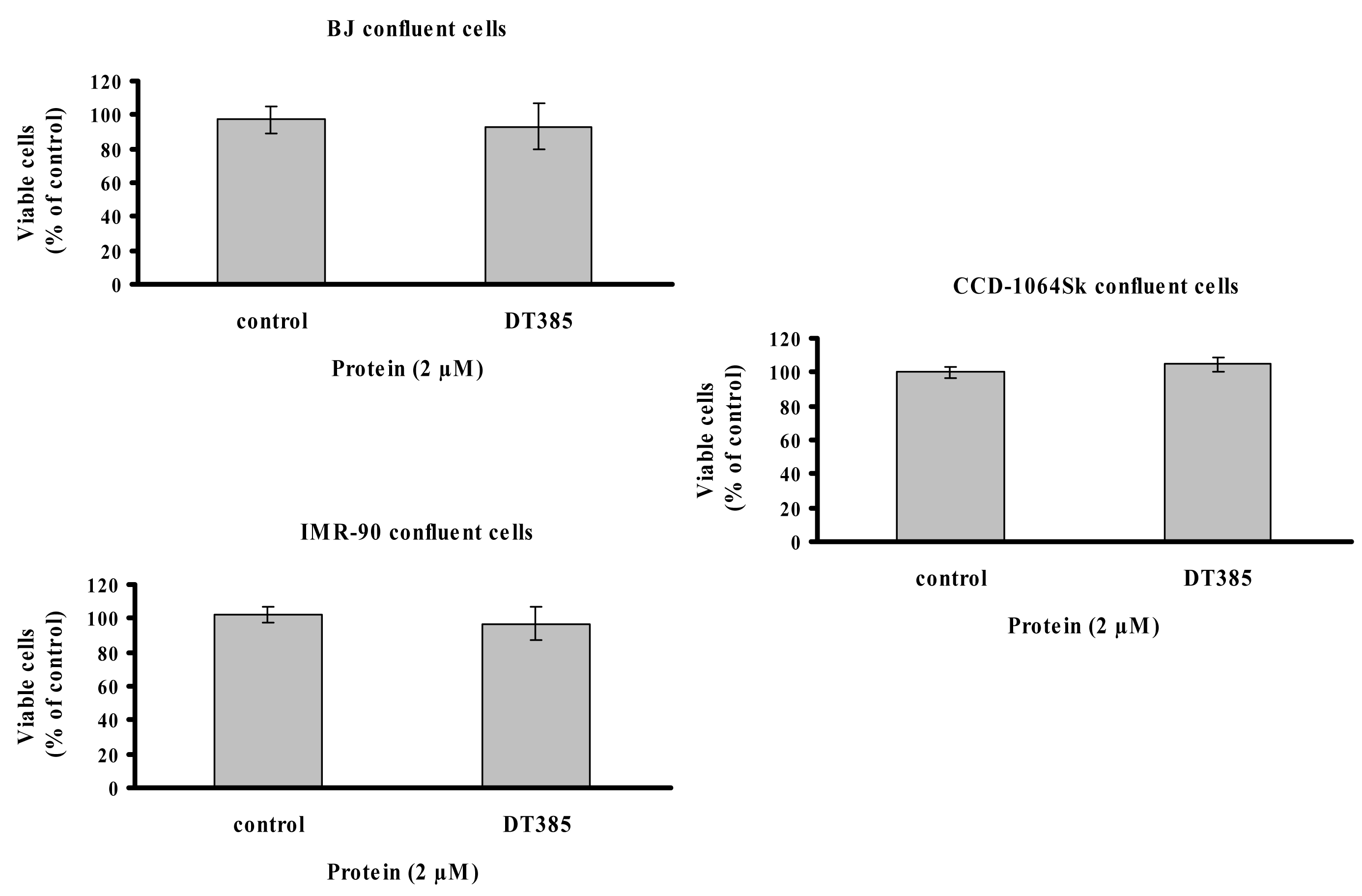

Supplement: Figure S2 — DT385 did not kill confluent cells. Cells were grew to confluent first, then incubated in fresh media and treated with 2 µM of DT385 for 3 days. Viable cell numbers after the three day incubation were quantified by the CellTiter 96 AQueous (Promega) assay. Cells without treatments were used as controls. Alternatively, PBS or control protein treatments were used as controls. Data are expressed as percent control response. Results are the mean ± S.D. of 3 experiments performed in duplicate (n = 6). (0.44 MB TIF) [file pone.0010498.s002.tif]

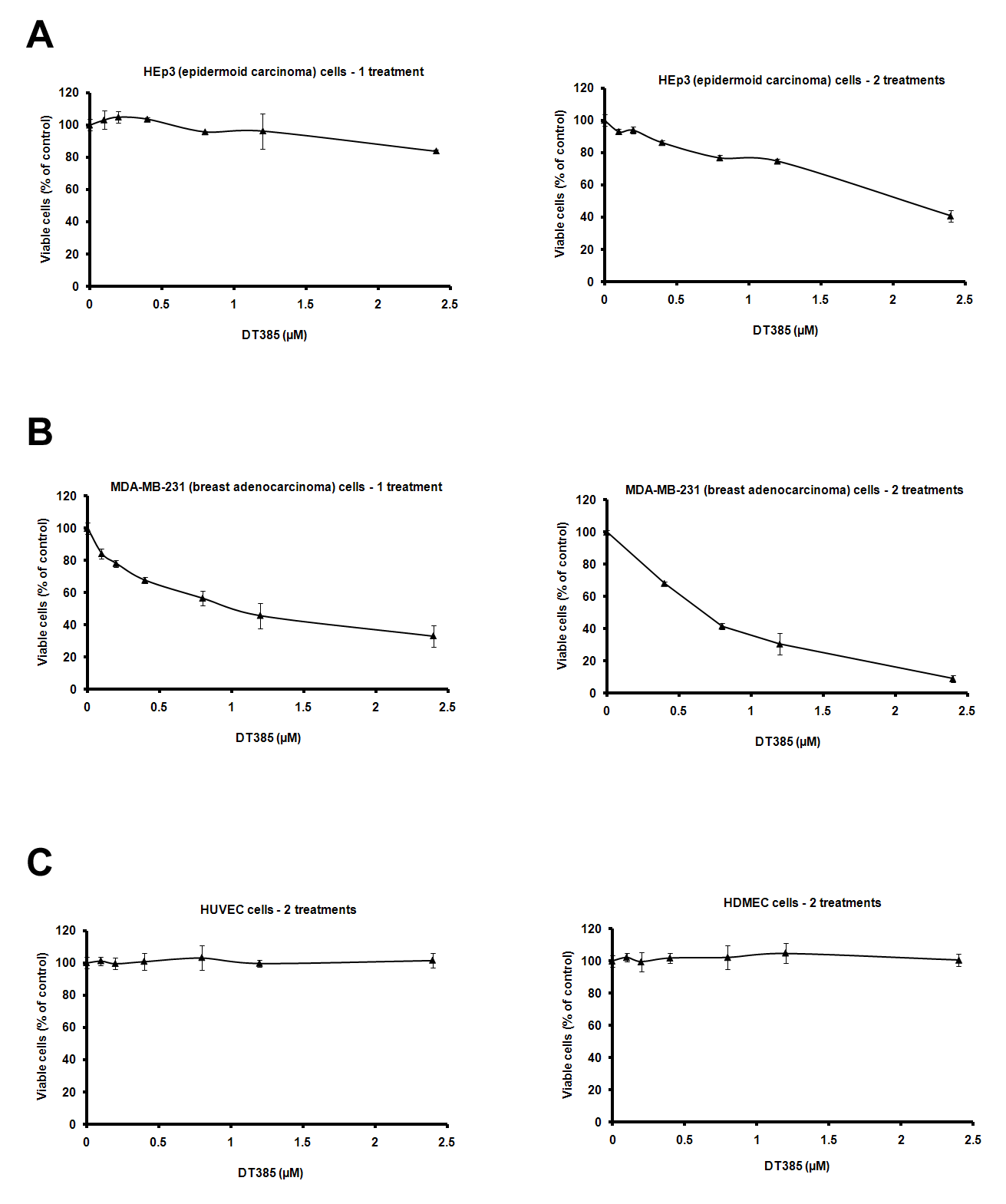

Supplement: Figure S3 — Two Applications of DT385 and DT-p22 Increased the Proliferation Inhibition Effect on Tumor Cells, but not on Human Endothelial Cell Lines. Cells were first treated with DT385 for 48 h, washed with PBS and then were further treated with a second application of DT385 for 48h (2 treatments) or simply incubated with DT385 for 4 days (1 treatment). Viable cells were then quantified by the CellTiter 96 AQueous (Promega) assay. A), Hep3, B), MDA-MB-231 cells were administrated DT385 at the indicated concentration. C), HUVEC or HdMEC cells were administrated 2 applications of DT385 at a concentration of 2.5 µM. PBS was used as a control treatment. Data are expressed as percent of control treatments. Results are the mean ± S.D of 2 experiments performed in triplicate (n = 6). (0.20 MB TIF) [file pone.0010498.s003.tif]

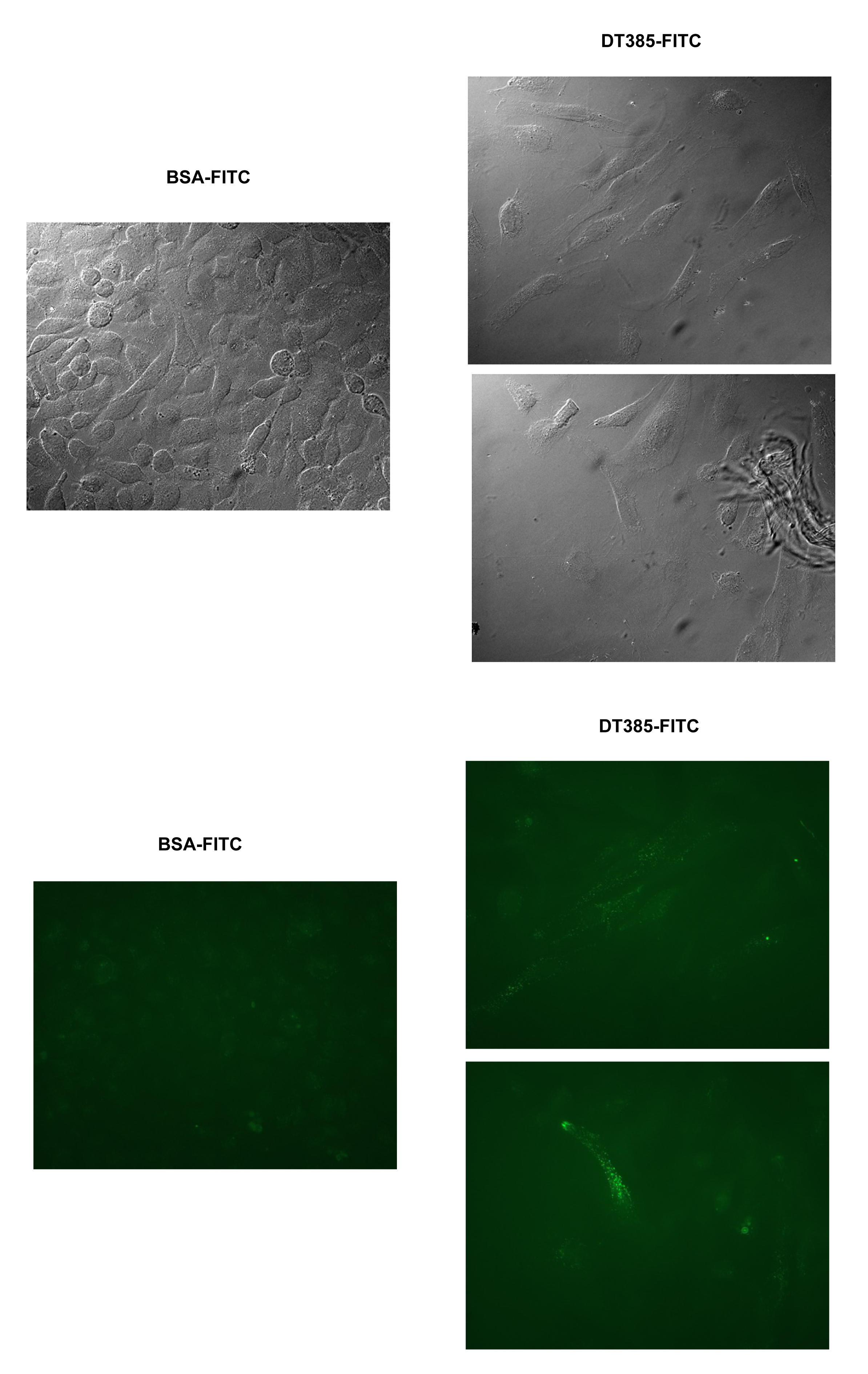

Supplement: Figure S4 — Internalization of DT385 by Cancer Cells. Cancer cells were grown in an 8-well chamber slide. FITC-labeled DT385 or BSA (DT385-FITC or BSA-FITC, 1 µM) was added to culture. The cells were observed and photographed with a Zeiss Axioplan II fluorescence microscope (Carl Zeiss, Germany) after 36 h. A), bright-field images. B), fluorescence images. (3.81 MB TIF) [file pone.0010498.s004.tif]

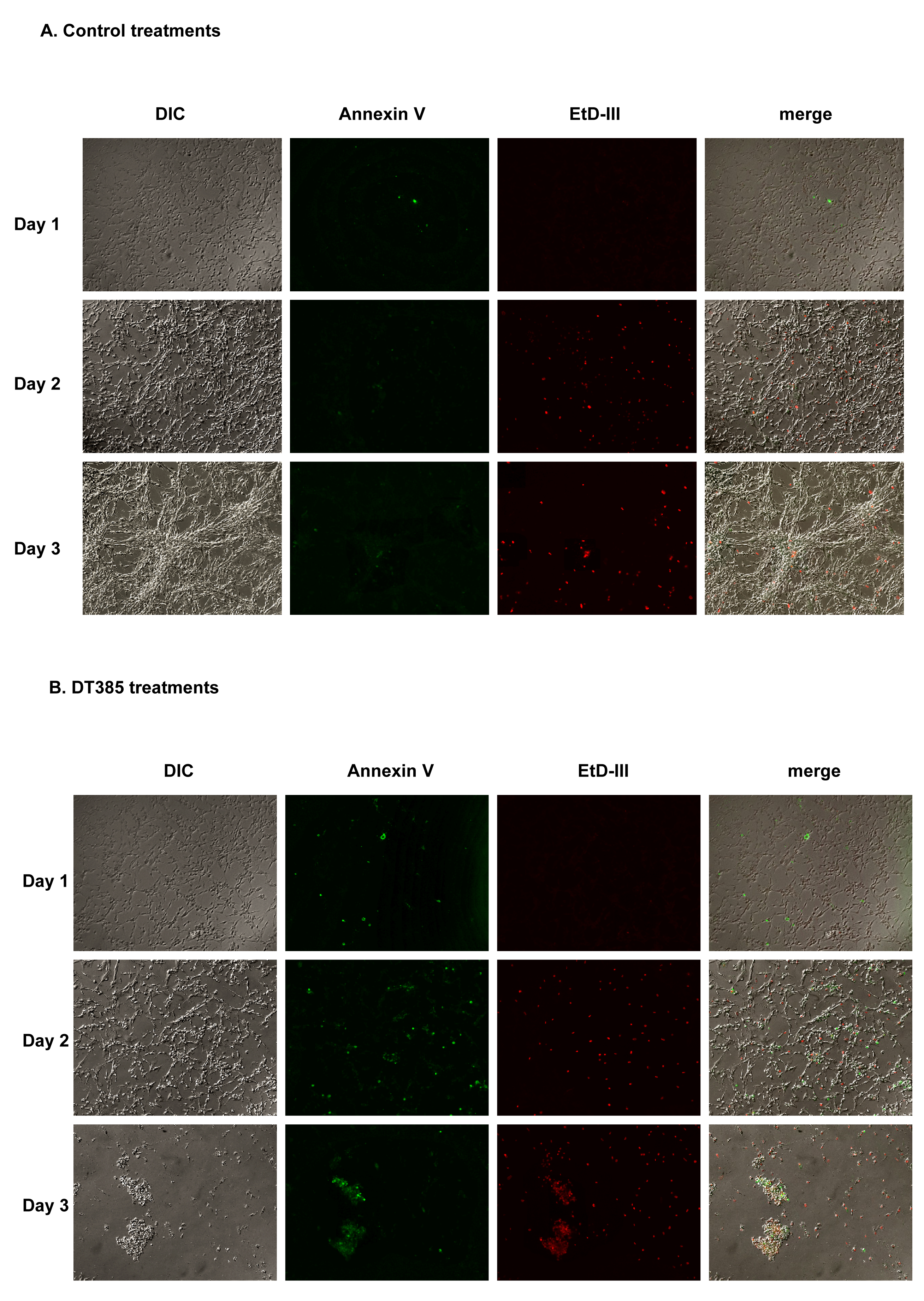

Supplement: Figure S5 — Time Course Assays of Apoptosis Caused by DT385. U-87 MG cells growing in an 8- well chamber slide were treated with 1.2 µM of DT385 (A) or control protein (B), respectively. Following treatments, cells were stained for apoptosis with FITC-labeled annexin V. Cell membrane integrity was evaluated with ethidium homodimer III (EtD-III) according to the manufacturer's instructions (Biotium, Inc). Staining images were photographed under a Zeiss Axioplan II fluorescence microscope (Carl Zeiss, Germany). Digital images were processed in Adobe Photoshop (Adobe Inc.). Representative images (100× magnifications) were shown. (7.69 MB TIF) [file pone.0010498.s005.tif]
